# Soil gas radon assessment and development of a radon risk map in Bolsena, Central Italy

**DOI:** 10.1007/s10653-014-9649-9

**Published:** 2014-09-20

**Authors:** G. Cinelli, L. Tositti, B. Capaccioni, E. Brattich, D. Mostacci

**Affiliations:** 2 Department of Biological, Geological and Environmental Sciences – Section of Geology, Alma Mater Studiorum University of Bologna, Piazza di Porta San Donato 1, 40126 Bologna, Italy; 3 Department of Chemistry «Giacomo Ciamician», Alma Mater Studiorum University of Bologna, Via Selmi 2, 40126 Bologna, Italy; 4 Laboratory of Nuclear Engineering, Via dei Colli 16, 40136 Bologna, Italy; 1 European Commission, Joint Research Centre (JRC), Institute for Transuranium Elements (ITU), Via E Fermi 2749, 21027 Ispra, Italy

**Keywords:** Vulsini Volcanic district, Soil gas radon, Geology, Radon risk map

## Abstract

Vulsini Volcanic district in Northern Latium (Central Italy) is characterized by high natural radiation background resulting from the high concentrations of uranium, thorium and potassium in the volcanic products. In order to estimate the radon radiation risk, a series of soil gas radon measurements were carried out in Bolsena, the principal urban settlement in this area NE of Rome. Soil gas radon concentration ranges between 7 and 176 kBq/m^3^ indicating a large degree of variability in the NORM content and behavior of the parent soil material related in particular to the occurrence of two different lithologies. Soil gas radon mapping confirmed the existence of two different areas: one along the shoreline of the Bolsena lake, characterized by low soil radon level, due to a prevailing alluvial lithology; another close to the Bolsena village with high soil radon level due to the presence of the high radioactive volcanic rocks of the Vulsini volcanic district. Radon risk assessment, based on soil gas radon and permeability data, results in a map where the alluvial area is characterized by a probability to be an area with high Radon Index lower than 20 %, while probabilities higher than 30 % and also above 50 % are found close to the Bolsena village.

## Introduction

Natural radioactivity is widely distributed in the lithosphere as well as in all the various environmental compartments as a result of nucleosynthesis and biogeochemical cycling, providing a large fraction of the background radiation dose to human population. It is mainly characterized by a small number of primordial radionuclides among which the most relevant ones are ^40^K and the members of the three natural radioactive families of ^238^U, ^232^Th and ^235^U. All the three families present an intermediate radon isotope which, due to the noble gas properties, can be easily remobilized from the mineral matrices where it is produced by radioactive decay. Radon isotopes of natural origin are, respectively, ^222^Rn (half-life *t*_1/2_ = 3.8 day), ^220^Rn (*t*_1/2_ = 55.6 s) and ^219^Rn (*t*_1/2_ = 3.96 s), in order of decreasing half-life and hence, on a first approximation, radiologic importance. The parent radionuclides of radon isotopes are present in all the crustal materials and their derivatives such as soil, industrial wastes coming from ore smelting, mineral extraction and industrial use, fossil fuel employment and cycles, waste recycling and building materials, from which they are released into the atmosphere (UNSCEAR 2008; Eisenbud and Gesell 1997).

Public exposure to natural ionizing radiation is largely due to radon. The estimated value of worldwide average annual exposure to the various components of natural radiation show that ^222^Rn contribution constitutes as much as 50 % of the overall radiation dose reaching values of about 1.15 mSv/year per capita (UNSCEAR 2008). As a general rule, radon exposure is largely due to its accumulation within confined environments with reduced or no air exchange, leading to the inhalation of potentially hazardous amounts of airborne alpha emitters both in the gaseous and in the particulate form with very well recognized epidemiological noxious effects (Porstendörfer 1994; UNSCEAR 2006; WHO 2008).

The cumulative effect of several factors, such as lithology, geomorphology, local/regional geotectonics and finally building materials and techniques as well as living habits (ventilation), lead to indoor radon accumulation and its inherent health risk.

Its transfer from the parent material into the atmosphere is controlled by a number of physical factors, such as porosity and degree of fracturing, temperature and pressure gradients, and moisture. Radon emitted from the ground surface or from materials of crustal origin in outdoor air is rapidly dispersed leading to low atmospheric concentration levels, while in confined environments, such as buildings, dwellings, tunnels, caves and mines, radon accumulates leading to potentially hazardous indoor concentration levels in the absence of mitigation actions.

The new Euratom Directive on Basic Safety Standards by the European Union published in January 2014 (EC 2013) presents several new aspects concerning natural radioactivity with respect to the 1996 edition, e.g., the radon action plan in which “…Member States shall establish a national action plan addressing long-term risks from radon exposures in dwellings, buildings with public access and workplaces for any source of radon ingress, whether from soil, building materials or water… Member States shall identify areas where the radon concentration (as an annual average) in a significant number of buildings is expected to exceed the relevant national reference level….”

In the past (EC 1996), those areas have been usually called ‘‘radon-prone areas’’,^1^ even if there was no authoritative definition of a radon-prone area. According to Bossew et al. (2013), “Qualitatively, this concept denotes areas where observed or expected values of a ^222^Rn-related variable are high with respect to reference values or with respect to the mean over the domain”. In general, radon-prone areas are identified according to two main strategies: (a) direct measurements of indoor radon concentration and (b) indirect methods including soil gas radon survey and gamma-dose assessment based on γ-spectrometry from laboratory, field and aerial surveys all implying the existence of a more or less known transfer factor from the crustal environment into the buildings (Dubois and Bossew 2006; García-Tavalera et al. 2013). Since each single methodology is usually not sufficient to the scope, hybrid approaches are often used suggesting the need for integrated information as an optimal tool for radon area classification. The process is finalized once geostatistical elaborations of the measured parameters have been suitably performed. For instance, maps based on indoor radon measurements and integrating geological information have been used in Great Britain (Miles and Appleton 2005) and Belgium (Cinelli et al. 2010), whereas maps based on soil gas measurements have been developed in Germany (Kemski et al. 2009) and Czech Republic (Barnet et al. 2008). At European level, the Radioactivity Environmental Monitoring (REM) group of the Joint Research Centre (JRC) of the European Commission started the European Atlas of Natural Radiation (EANR) (De Cort et al. 2011) some years ago. The European indoor radon map (EIRM) displays annual mean indoor radon concentrations in ground floor rooms of dwellings on a defined reference grid with a resolution of 10 km × 10 km (Gruber et al. 2013b). In parallel to this effort, a European Geogenic RadonMap (EGRM) is under development by the JRC: This map aims to display a quantity closer to geogenic hazard, i.e., which measures “what earth delivers” in terms of radon irrespective of anthropogenic factors and temporally constant over a geological timescale (Gruber et al. 2013a).

In order to assess the radon risk in buildings in a given area on the basis of soil radon or of radon-related variables, it is necessary to set up indexes accounting for the effective radioactivity levels as well as for the physical properties of solids through which radon transfer occurs. This approach based on radon potential assessment and on the use of Radon indexes such as those authoritatively introduced by Barnet and coworkers in the Czech Republic (Barnet et al. 2008) first and presently extending to the other European countries, allows to predict the indoor radon in new buildings and to estimate the radiological risk from this relevant but ubiquitous radiation source.

This work presents the results of an investigation concerning NORM^2^ distribution in one of the areas with the highest natural background radiation in Italy. In particular, the study concerns the Vulsini Volcanic district in northern Lazio, a well-known quaternary volcanic area subject of a previous paper recently published by the authors aimed at the assessment of both the regional NORM level and distribution as related to the specific magmatic processes and of the associated radon risk (Capaccioni et al. 2012). In this area, volcanic products have relevant U, Th and K contents ranging, respectively, from 6 to 32 ppm, from 31 to 120 ppm and from 0.7 to 8 % by weight. Beside the lithological and radiological assessment in connection with the different Vulsinian volcanic rocks, this paper includes a limited, but significant number of indoor radon measurements covering both historical and modern buildings in order to collect preliminary information on radon exposure in this region. Markedly, high values were recorded in buildings (46–3,269 Bq/m^3^) and in cellars (30,000 Bq/m^3^) pointing at the need of a more systematic investigation related to the territory rather than to specific situations and therefore reflecting radon risk areal distribution for the local population with a more rigorous approach. Moreover, Capaccioni et al. (2012) pointed out the strong relationships between the high indoor radon level and the local rock formations able to affect both indoor radon buildup, as a part of the local landscape, and building material, especially tuffs, whose use in this area is historically recognized since ancient times. In spite of the evidence concerning the high radiation background in the investigated region, the relationships between rocks radioactivity and soil gas radon in a given area is not obvious, since many factors and processes may contribute to influence the latter. In particular, soil gas radon depends on uranium–radium concentration in the residual mineral fraction, the degree of remobilization of members of the radioactive families by weathering and/or post depositional chemical processes, porosity, faulting and advection in case of secondary magma degassing from a deep source. However, since the major source of indoor radon is in the soil and in the bedrock surrounding and under the buildings, our investigation on the Vulsini district was extended to the collection of data on soil gas radon in this region.

The present paper is therefore focused on the results obtained from a campaign of soil gas radon measurements mainly carried out in the urban area of Bolsena, the most important town of the Vulsinian volcanic district. Its main aims are integrating previous information on this high background radiation area, evaluating local levels of soil gas radon, creating both a soil gas radon map and a conjunct radon risk map integrating soil gas radon data, geological information and radiation protection issues, and, finally, the identification of radon-prone areas, as suggested by the International Commission for Radiological Protection (ICRP) and the EU Council Directive 96/29/EURATOM (1996) and the new Euratom Directive on Basic Safety Standards published in January 2014 (EC 2013).

## Geological setting

The Vulsini Volcanic district (Nappi et al. 1995; Capaccioni et al. 2012), which was active in the period 570–127 ka (Gillot et al. 1991), is located in the northern part of the Quaternary potassic volcanic belt of the Roman Magmatic province (central-southern Italy). Four volcanic complexes have been recognized in the evolution of the district: Paleo-Bolsena, Bolsena, Latera and Montefiascone (Nappi et al. 1991). They developed with alternating effusive and explosive eruptions, with Strombolian, Plinian and Ignimbrite-forming phases. Volcanic products are widely differentiated including leucite, basanite and shoshonite magmatic suites (Nappi and Valentini 2005).

The Bolsena volcanic complex mainly took place in the eastern part of the Vulsini district after an extensive phase of subsidence which affected the final activity of the Paleo-Bolsena volcanic complex, responsible for a thick sequence of volcano-lacustrine deposits within the Bolsena caldera (Nappi and Valentini 2005). The Bolsena volcanic complex developed during two eruptive cycles (Nappi and Marini 1986). The first one starts with an initial effusive and/or Strombolian phase and ends with a minor explosive and effusive phase. The second cycle also consists of a moderate initial effusive and/or Strombolian phase but ends with a large, paroxysmal explosive activity, producing a widespread tuff deposit. In details, the oldest products of Bolsena complex are represented by leucite-bearing lava flows outcropping in the N and E sectors. The final phase of the first cycle is characterized by pyroclastic falls, small scale pyroclastic flows, welded tuffs and several outward and inward trachytic lava flows. Phases of volcanic-tectonic collapses took place after this moderate explosive to effusive activity and part of the NE Bolsena caldera was outlined by complex faulting. N–S and NNW–SSE lines of weakness formed throughout the E side of the present Bolsena Caldera and high potassic series magmas were tapped. The leucite-bearing lava flows and scoria cones associated with this activity can be considered as the initial phase of the second Bolsena cycle. On the contrary, the final stage of the Bolsena complex gives rise to the pyroclastic sequence of the Orvieto-Bagnoregio Ignimbrite (333 ka, Nappi et al. 1991).

**Fig. 1:**
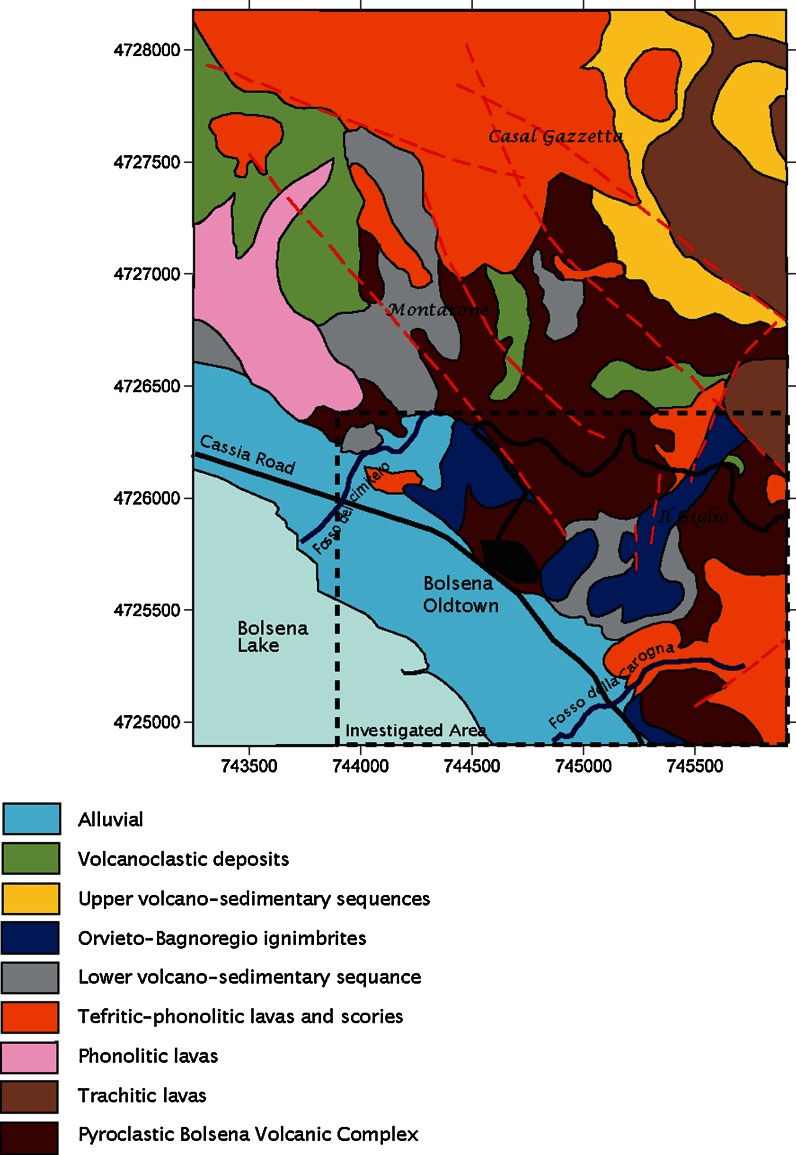
Geological sketch map of the area around Bolsena village (after Renzulli 1988, modified)

The detailed geological formations present around Bolsena village are shown in Fig. 1.

As outlined in a previous paper (Capaccioni et al. 2012), concentrations of natural radionuclides in local volcanic outcrops are rather high compared to average Earth’s crust substantially enhancing the regional radiation background. In particular, uranium and thorium concentrations, typically high in this kind of magmatic lithologies, have been found to range from 80 to 394 Bq/kg and from 126 to 487 Bq/kg, respectively (Capaccioni et al. 2012; Cinelli 2012). This agrees with the high indoor radon detected (ibidem) and conducive of high soil gas radon supported by both the local mineral matrices as well as from faulting and degassing as the declining phase of the secondary volcanic activity.

## Instruments and methods

### Data

**Fig. 2:**
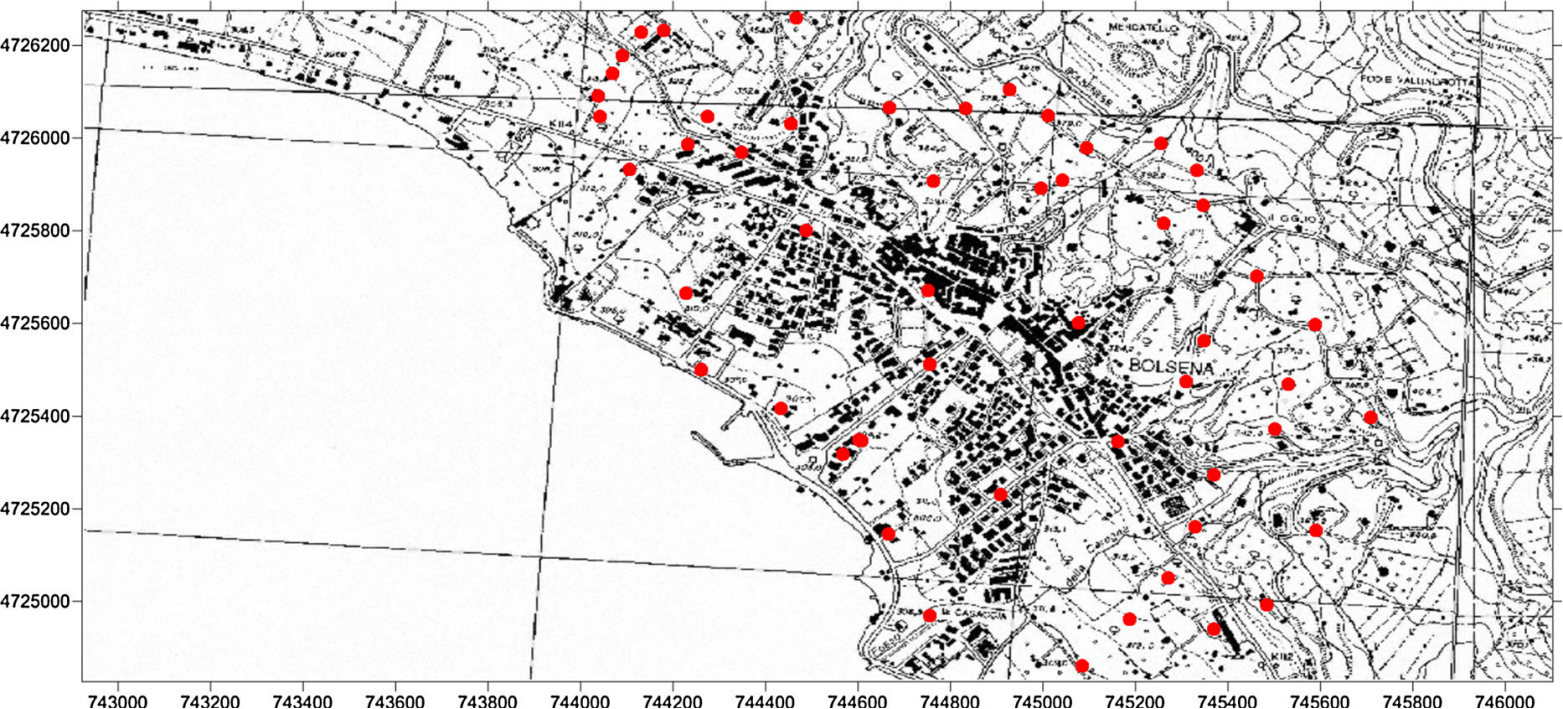
Locations in and around Bolsena where soil gas radon concentrations are measured; the *red points* indicate the measurement points

A soil gas radon campaign was carried out in March 2011 covering an area of about 2 km^2^ with 63 sampling stations distributed within the urban area of Bolsena and including part of its outskirt, following a square grid-based sampling design (see Fig. 2), with cell sizes about 100 m. All the stations occupied were georeferenced following the WGS 84 (UTM 32T) geographic coordinate system (Fig. 2) using a GPS (Garmin).

Soil gas radon was measured by coupling a RAD-7 radon monitor (Durridge Co.) to a standard soil probe. The soil gas probe was inserted down to an approximate depth of 60–70 cm where in this area you find the hard rock basement, so it was not possible to insert the probe to the suggested depth of 1 m (Barnet et al. 2008). Briefly, the instrument works by circulating air from the ground through a membrane filter to retain airborne particulate matter containing radon progeny and through desiccant (drierite) to prevent radon artifacts during measurements. Purified air from soil interstices is hence pumped inside the RAD-7 measuring chamber where radon gas is detected through α-decay of its daughter ^218^Po therein produced. In order to measure soil gas radon, the instrument was operated in sniff mode (RAD-7 2009). Since secular equilibrium between ^222^Rn and ^218^Po is reached in about 15 min, a single measurement must have an average duration of 25–30 min with readings taken every 5 min. The measurement is terminated when the relative difference between two consecutive readings is lower than 15 %. The final result is estimated as the average of the last two readings.

Eight soil samples, 1 kg each, were collected to estimate permeability, a parameter required by the radon risk modeling suggested by Barnet et al. (2008). It was possible to derive a rough estimate of the permeability very easily from the weight percentage of fine fraction (<63 μm). Soils with the weight percentage of the fine fraction <15 % were designed as high permeable soils, in the range 15–65 % as medium permeable and in the case of the fine fraction above 65 % as low permeable ones (Barnet et al. 2008).

Sieve analysis was carried out to determine the weight percentage of fine fraction. A wet-sieving process has been used in which the material is mixed with water until it becomes a suspension which is placed on the sieve (mesh size: 63 μm). Above the sieve, a water spray nozzle is placed which supports the sieving process additionally to the sieving motion. The rinsing is carried out until the liquid which is discharged through the receiver is clear. Sample residues on the sieves are dried at 105 °C (until the weight remained constant), and the fraction <63 μm can be calculated.

### High-resolution γ-spectrometry

Soil samples, crushed and sieved at 2,000-µm mesh, were analyzed by γ-spectrometry with a p-type coaxial Hyper Pure Germanium crystal detectors (HPGe), with an energy range 0–2,000 keV. The detector has a relative efficiency of 38 % and a resolution (FWHM) of 1.8 at 1,330 keV, respectively. The system was calibrated for energy and efficiency using a multiple nuclide source (QCY48, Amersham) in a jar geometry (diameter: 56 mm; thickness: 10 mm). Spectra were analyzed with the GammaVision-32 software (version 6.07, Ortec). Quantitative analysis on samples was carried out by subtracting the spectrum of water in the same geometry, while uncertainty on peaks (k = 1, 68 % level of confidence) was calculated propagating the combined error over the efficiency fit previously determined with the counting error. Minimum detectable activity was calculated making use of the Traditional ORTEC method (ORTEC 2003) with a peak cut-off limit of 40 %.

^238^U and ^232^Th were determined using the emissions of their radioactive descendants ^226^Ra and ^228^Ac. The correction of the ^226^Ra peak at 186 keV was carried out assuming a secular equilibrium between ^226^Ra–^238^U and natural ^235^U/^238^U isotopic ratio (Gilmore 2008). Under these two hypotheses, the ^226^Ra peak was corrected by dividing by 1.7337. The results were also checked according to the method, described by De Corte et al. (2005), based on the correction of the peak at 186 keV for the contribution of ^235^U using ^234^Th peak at 63.3 keV, assuming natural ^235^U/^238^U isotopic ratio and ^238^U–^234^Th in equilibrium. The correction of ^234^Th peak at 63.3 keV for the ^232^Th peak at 63.8 keV has been made using the ^228^Ac peak at 338.3 keV. The two methods led to very close corrected activity concentrations of ^226^Ra, with differences <5 %.

Conversion from specific activity (Bq/kg) to bulk elemental weight fraction was obtained with the following conversion factors (Stromswold 1995):

- 1 % K = 309.7 Bq/kg
- 1 ppm U = 12.35 Bq/kg
- 1 ppm Th = 4.072 Bq/kg.

To reach appropriate counting statistics, samples were counted for 80,000 s. Certified reference materials (DH-1a and UTS-3, CANMET) were used to verify the quality of the measurements.

### Geostatistics

Geostatistics offers a way to describe the spatial continuity of natural phenomena and provides adaptations of classical regression techniques to take advantage of this continuity (Isaaks and Srivastava 1989). It is thus natural that this collection of methods and tools was applied to analyze the spatial structure of soil gas radon concentration (Dubois and Bossew 2006). Kriging is the most common estimation procedure used in geostatistics (Langley 1971). It predicts unknown values using known values and a variogram model. By means of the variogram, an important component of the Kriging method, the spatial correlation between data is measured. The software Surfer11 (SURFER 2012) was used to study the spatial structure.

### Radon index

The geogenic radon potential is the essential parameter describing the subsurface as the main source for indoor radon independently on the building characteristics (Kemski et al. 2001); radon production and radon supply are the essential criteria for evaluating the geogenic radon potential. A way to define the radon potential is the Radon Index (RI) based on multivariate cross-tabulation (Gruber et al. 2013a). In this approach, scores are assigned to combinations of input quantities; the resulting RI is a categorical-ordinal quantity, i.e., in ordered classes as (I, II, III, IV) or (low, medium, high). The RI has been defined in this way in several countries such as the USA (EPA 1993), the Czech Republic (Barnet et al. 2008) and Germany (Kemski et al. 2001).

The radon risk classification in Czech Republic is based on the assessment of two main parameters: the soil gas radon (^222^Rn) concentration and the permeability of soil and rock for gasses. If the numerical value of permeability is not available (as in our case), it is sufficient to estimate it as low, medium or high and the radon index of the building is assessed using the classification reported in Table 1 (Barnet et al. 2008) .^3^

**Table 1:** Radon index (risk) assessment (Barnet et al. 2008)

Radon index (RI) category	Soil gas radon concentration C (kBq/m^3^)		
Low	c < 30	c < 20	c < 10
Medium	30 ≤ c < 100	20 ≤ c < 70	10 ≤ c < 30
High	c ≥ 100	c ≥ 70	c ≥ 30
	Low	Medium	High
	Permeability		

### Radon mapping methodology

Geostatistical elaboration for a soil gas radon and a radon risk mapping was carried out based on the following criteria:

- The area was subdivided into square cells with a surface of 100 m × 100 m, each cell being characterized by a coordinate pair associated to its center;
- Geological information available in Renzulli map (Renzulli 1988) was attributed to the center of each square cell through available georeferencing in GIS system;
- Choosing the variogram estimator in the software Surfer 11 (SURFER 2012), an experimental variogram is computed for the total database and for each geological group;
- Calculation of mean radon concentration was referred to the cell center. Mean radon concentration was predicted applying Kriging to the experimental values (or ln-transformed data) considering only the nugget effect in the variogram model which makes Kriging practically equivalent to the simple moving average. In the algorithm, the following values of constraining conditions were chosen:
  15: the maximum number of data to be used to calculate the mean at the cell center;
  10: the minimum number of data to be used to calculate the mean at the cell center; fewer data can lead to doubtful results;
  500 m: the distance (radius) from cell center that the code looks up to find data points when calculating the mean at the cell center;
- Radon risk mapping required the calculation of a Radon Index (RI), which in the present work, was estimated following Barnet methodology (2008) based on the soil gas radon concentration and permeability given as low, medium or high. In the hypothesis of locally normal or log-normal distribution for soil gas radon data, the probability to be in an area with high Radon Index (P (hRI)) has been calculated using the following expression:
  1 $$P(hRI) = \int\limits_{{C_{p} }}^{\infty } {\exp \left( { - \frac{{(x - \bar{x})}}{{2 \times \sigma_{g} }}} \right) \times \frac{1}{{\sigma_{g} \times \sqrt {2\pi } }}} \delta x$$
  where $\bar{x}$ is the mean of soil gas radon concentration calculated at the cell center in the case of normal distribution or the logarithmic mean in case of lognormal distribution; σ_g_ is the standard deviation of the group of data considered in the case of normal distribution or the logarithmic standard deviation in case of lognormal distribution; C_p_ is the soil gas radon concentration threshold, given the permeability class, to be in the high radon index category (Table 1) if the distribution is normal or the ln (Cp) if the distribution is lognormal.

## Results and discussion

Soil gas radon data from the Bolsena area presented in this work range between 7 and 176 kBq/m^3^ (Table 2). Table 3 reports a comparison of the data collected in the Bolsena area with data from other locations including regional, national and international places among those available from the literature. It can be observed that Bolsena mean data are intermediate with respect to other situations reported in Table 3. It can be seen that the arithmetic mean is twice the mean for Italy, while it appears very close to data from Ciampino–Marino (the Alban Hills), i.e., at the southernmost tips of the Latium Quaternary Volcanic complex, which is characterized by the same potassic and ultrapotassic volcanism as the Volsini Volcanic district (see Capaccioni et al. (2012) for details on the high radiation background in this area).

**Table 2:** Main statistics parameters of soil gas radon concentrations (kBq/m^3^) and the ln-transformed data (Bq/m^3^) considering the full database and the geological groups separately

	Total	Volcanic products	Alluvial	Ln-transformed data		
				Total	Volcanic products	Alluvial
Valid N	63	36	27	63	36	27
Mean	56.2	66.9	42.0	10.7	10.9	10.4
Median	52.4	66.2	36.9	10.9	11.1	10.5
Minimum	7.0	9.3	7.0	8.9	9.1	8.9
Maximum	176.0	176.0	90.9	12.1	12.1	11.4
1. Quartile	29.6	42.9	24.2	10.29	10.67	10.09
3. Quartile	75.6	86.3	63.2	11.23	11.36	11.05
SD	33.5	35.6	24.6	0.72	0.66	0.71
Skewness	0.93	0.81	0.49	−0.82	−1.13	−0.7
Excess Kurtosis	1.47	1.27	−0.73	0.33	1.53	0.048
*p* value S–W test^a^	0.0084	**0**.**1886**	**0**.**1257**	0.0055	0.0086	**0**.**0942**
*p* value *t* test		0.0028			0.0054	

^a^Shapiro–Wilk test

**Table 3:** Comparison of soil gas radon concentration (kBq/m^3^) obtained in the present work with those obtained in Italy and in the Czech Republic published in the literature

^222^Rn (kBq/m^3^)	Mean	Median	Min	Max	SD	LQ	UQ
Italy^a^	26.6	13.7	0.4	1,200	39.5	5.5	32.6
Volcanic areas^a^	43.4	28.5	0.4	393.3	45.0	12.2	59.9
Bolsena (this work total database)	56.2	52.4	7.0	176.0	33.5	29.6	75.6
Ciampino–Marino^b^	52.0	30.5	1.5	367.3	47.2	23.3	64.8
Austria^c^	75		40	600			
Czech Republic^c^	28.1	–	1	1,664	40.4	–	–
Protezozoic-Paleozoic volcanites^d^	28.5	21.7	–	233.8	28.7	10.9	37.1
Tertiary volcanites^c^	21.14	16.20	–	197.2	25.8	6.90	26.0
France^c^	58.0			598	42.0		
Germany^c^	55.0		<5	>1,000			
Igneus rocks^c^	110.0		<5	>1,000			

^a^Beaubien et al. (2003)

^b^Etiope and Lombardi (1994)

^c^Dubois (2005)

^d^Barnet et al. (2008)

**Fig. 3:**
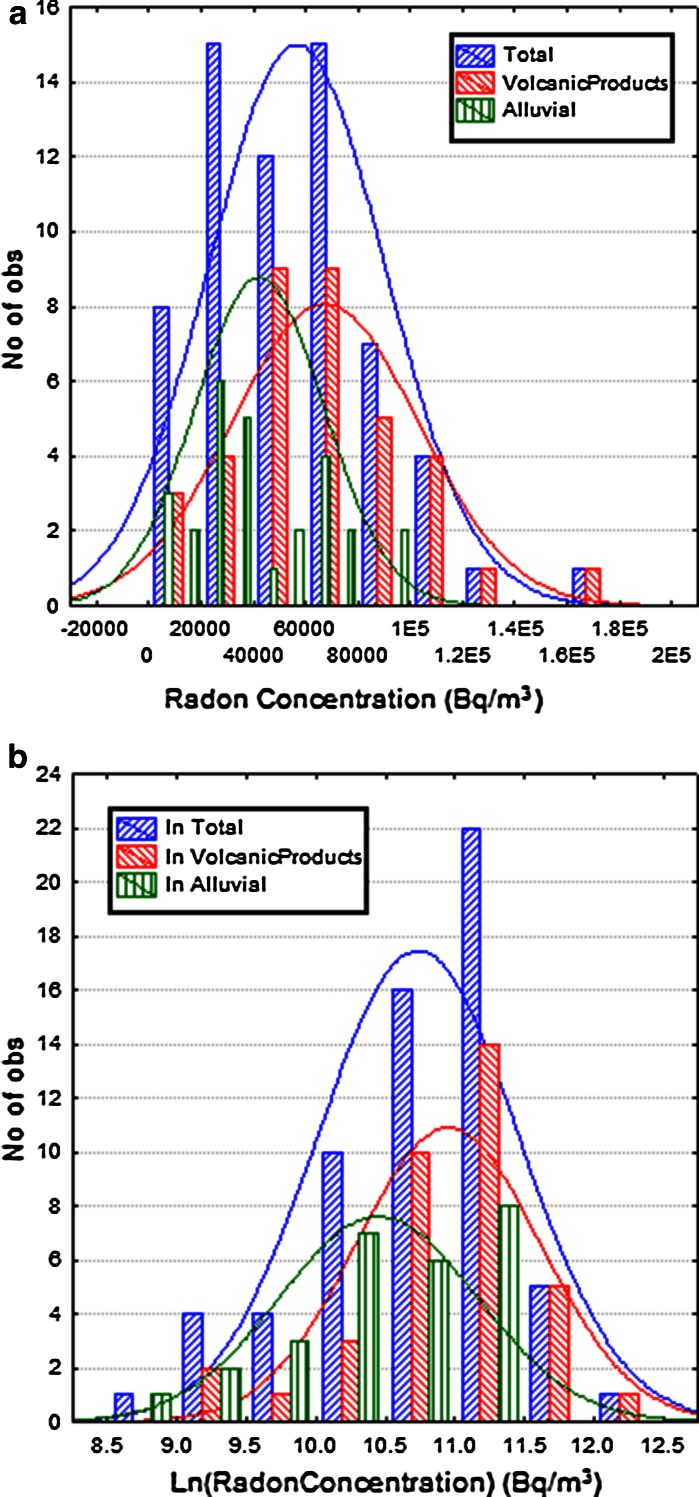
**a** Histogram and normal distribution fit (*continue line*) considering total database (*blue color*) and the two geological groups separately (*green* *alluvial*, *red* *volcanic*) and **b** considering the ln-transformed data

Figure 3a, b depicts the soil gas radon data, and the ln-transformed data obtained from Bolsena survey, respectively. The data reported in Fig. 3a shows two peaks, respectively, at about 30 and 60 kBq/m^3^ which may suggest a bimodal distribution. This is expected to be observed in connection with two distinct geochemical matrices with different uranium–radium concentration levels: Soil gas radon detected in the two subsets will reflect both parent lithologies described by Nappi et al. (1982) and/or possibly different remobilization processes due to chemical weathering. This finding seems to be in agreement with the observations given by Cinelli (2012) who showed that Volsinian volcanic lithology in this area are typically characterized by high NORM content. In particular, according to the similarity observed between the soil gas radon from Bolsena and Ciampino–Marino previously mentioned, it seems reasonable to associate the lower level soil gas radon peak to the alluvial component and the higher one to the volcanic products, respectively, depicted in green and red in Fig. 3a and b.

Soil gas radon data distribution was therefore solved by decomposing the dataset into two independent components which belong to the two different geological units (volcanic products and alluvial), according to the thematic geological map (Fig. 1) and the location of sampling points (Fig. 2). Statistical parameters for the two soil gas radon components are reported in Table 2; the *t* test shows that the two subsets of data are not statistically compatible (*p* < 0.05).

**Fig. 4:**
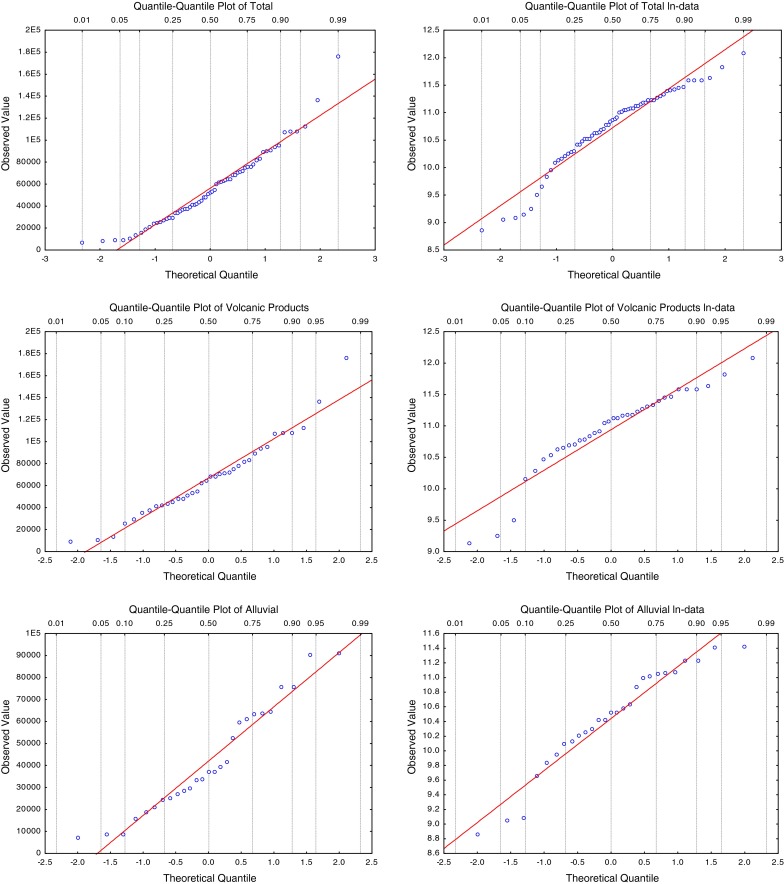
On the *left* normal quantile–quantile plots for soil gas radon concentrations (C) measured in the total, volcanic products and alluvial areas. On the *right* normal quantile–quantile plots for ln-transformed soil gas radon concentrations (C) measured in the total, volcanic products and alluvial areas

The hypothesis of normality and log normality was verified for the total dataset and for each of the two distinct subsets using the Shapiro–Wilk test (Table 2). It shows that the normality is verified for both groups of data belonging to the two different geological units, while the log normality is verified for only one. On the basis of the statistical parameters (Table 2), the histograms (Fig. 3a, b) and the q–q plots (Fig. 4), the two subsets belonging to the two different geological units could be approximated with a normal distribution. The model distribution is used to evaluate the percentage of cases higher than *Cp* (Eq. 1). In this context, the bad fits to low-concentration data, or to data above *Cp*, have no consequence. The important point is that the model should correctly predict the quantile corresponding to *Cp*, which is reasonably achieved by the normal distribution.

### Comparison between experimental results and modeled soil gas radon data

**Fig. 5:**
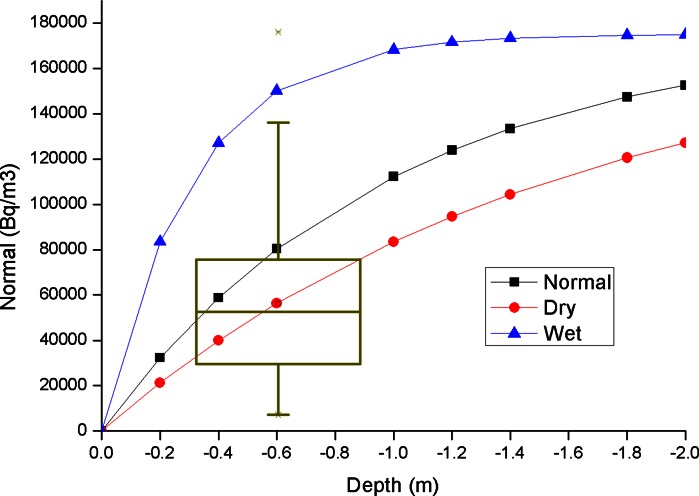
Calculated diffusive profile in an ideal soil, using ^226^Ra activity and emanation coefficient from average soil samples. *Box–whisker* plots superimposed to modeled profiles represent the experimental soil gas radon data described in this work: The *box* ranges between 25/75th percentiles and the *whisker* coefficient is 1.5, while crosses indicate outliers

For the sake of completeness, soil gas radon data measured in Bolsena were compared with the value estimated applying a soil-pore diffusion model proposed by Nero (1988) on the basis of the parent ^226^Ra measured in a small set of soil samples by high-resolution γ-spectrometry. The model includes both diffusive and advective transport of radon and admits a simple analytical solution under the hypotheses of semi-infinite homogeneous isotropic soil, with a one-dimensional vertical steady-state transport. For a purely diffusive transport, assuming radon concentration to be negligible in free air, the models predicts a simple concentration profile versus depth in the soil:

2 $$C(z) \approx \left( {1 - e^{{z\sqrt {{\raise0.7ex\hbox{${\lambda_{Rn} }$} \!\mathord{\left/ {\vphantom {{\lambda_{Rn} } {D_{e} }}}\right.\kern-0pt} \!\lower0.7ex\hbox{${D_{e} }$}}} }} } \right) \cdot C( - \infty )$$

where *C* is the radon concentration in pores (Bq/m^3^), *D*_e_ the effective diffusion coefficient (m^2^/s), λ_Rn_ the decay constant of radon (s^−1^), and *C*(−∞) = *G*/λ_Rn_, G being the volumetric radon generation rate in the soil pores (Bq m^−3^ s^−1^) defined as follows

3 $$G = A_{Ra} \cdot \rho \cdot f \cdot \lambda_{Rn} \cdot \left( {\frac{1 - \varepsilon }{\varepsilon }} \right)$$

where A_Ra_ is the activity concentration (Bq kg^−1^) of ^226^Ra, *f* the emanation factor (dimensionless), defined as the ratio between the radon activity emanating in the air-filled pores to the total radon production in the material, and ρ is the bulk density of the material (kg m^−3^). The important parameters were set equal to: ρ 2,650 kg/m^3^, ε 0.434, *D*_e_ 2 × 10^−6^ m^2^/s for “normal” soil, 5 × 10^−6^ m^2^/s for “dry” soil and 2 × 10^−7^ m^2^/s for “wet” soil as proposed in Nero (1988), respectively. The ^226^Ra activity was fixed equal to 142 Bq/kg, the average value of the activity concentrations measured in soil samples (132, 127, 169 Bq/kg) by γ-spectrometry analysis. The emanation coefficient was fixed equal to 0.33, measured by gamma spectrometry ad 1 minus the ratio between the mean specific activity of radon decay products ^214^Pb and ^214^Bi w.r.t. the parent ^226^Ra (mean value of analyzed soil samples 0.25, 0.27 and 0.47). The concentration profile reported in Fig. 5 shows that the theoretical soil radon values, calculated in an ideal soil where only the diffusive transport is considered, at depth of 60–70 cm, are in good agreement with the average value of the measured data, i.e., about 56 kBq/m^3^ (Table 2). However, the model is too much schematic to be used to replace soil radon measurements.

### Geostatistical elaboration and soil gas radon concentration mapping

**Fig. 6:**
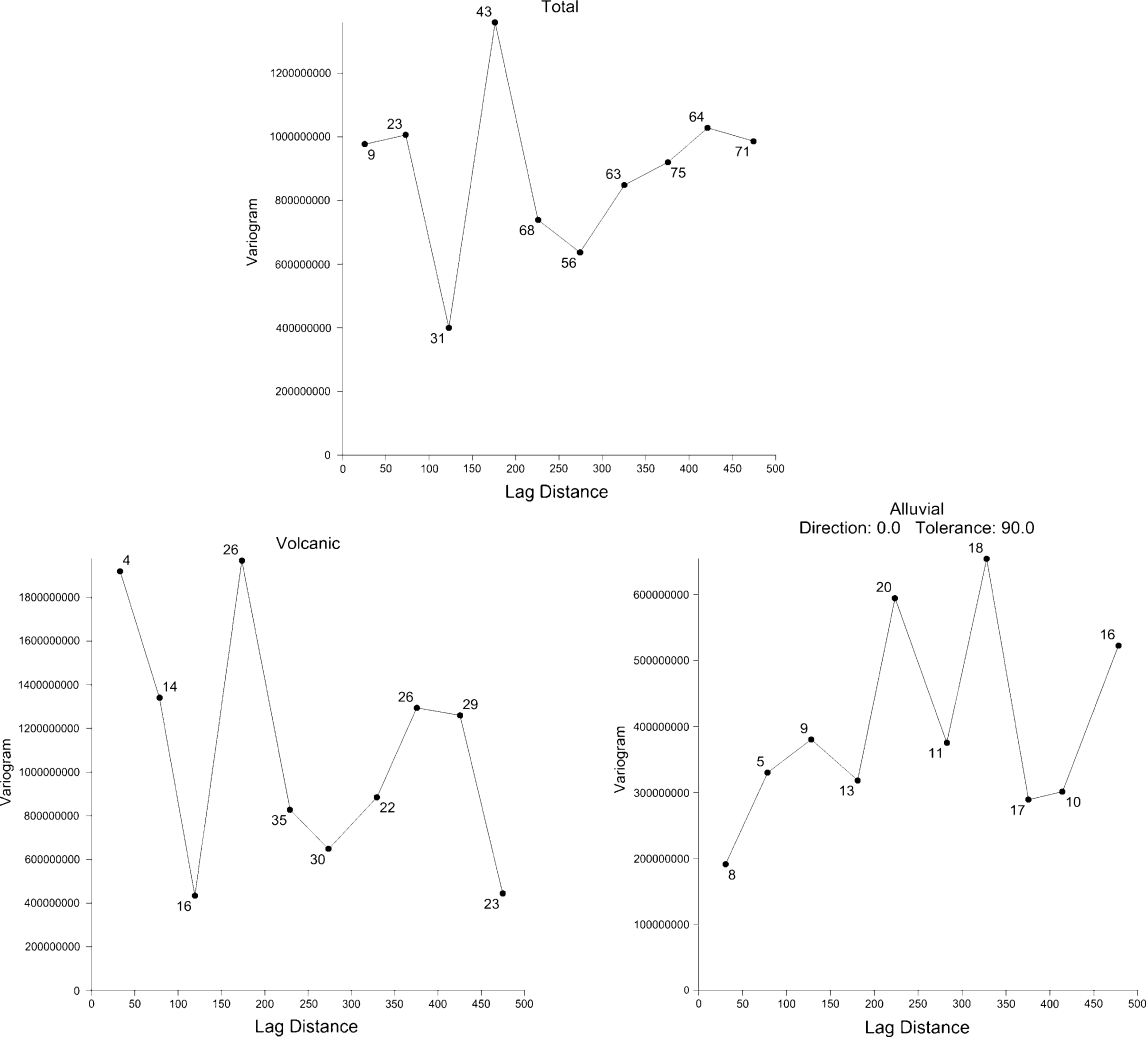
Empirical variograms of Rn concentration considering the total database and the two geological subgroups: volcanic and alluvial

Soil gas radon variograms have been elaborated both for the full database and for the two distinct geological groups previously extracted. They show significant statistical fluctuations; therefore, the choice of the variogram model is not obvious (see isotropic variograms in Fig. 6). Also anisotropic experimental variograms calculated using an angular tolerance of 30° show no variation with direction. As no spatial correlation is seen, the choice is oriented to the constant variogram model (pure nugget).

**Fig. 7:**
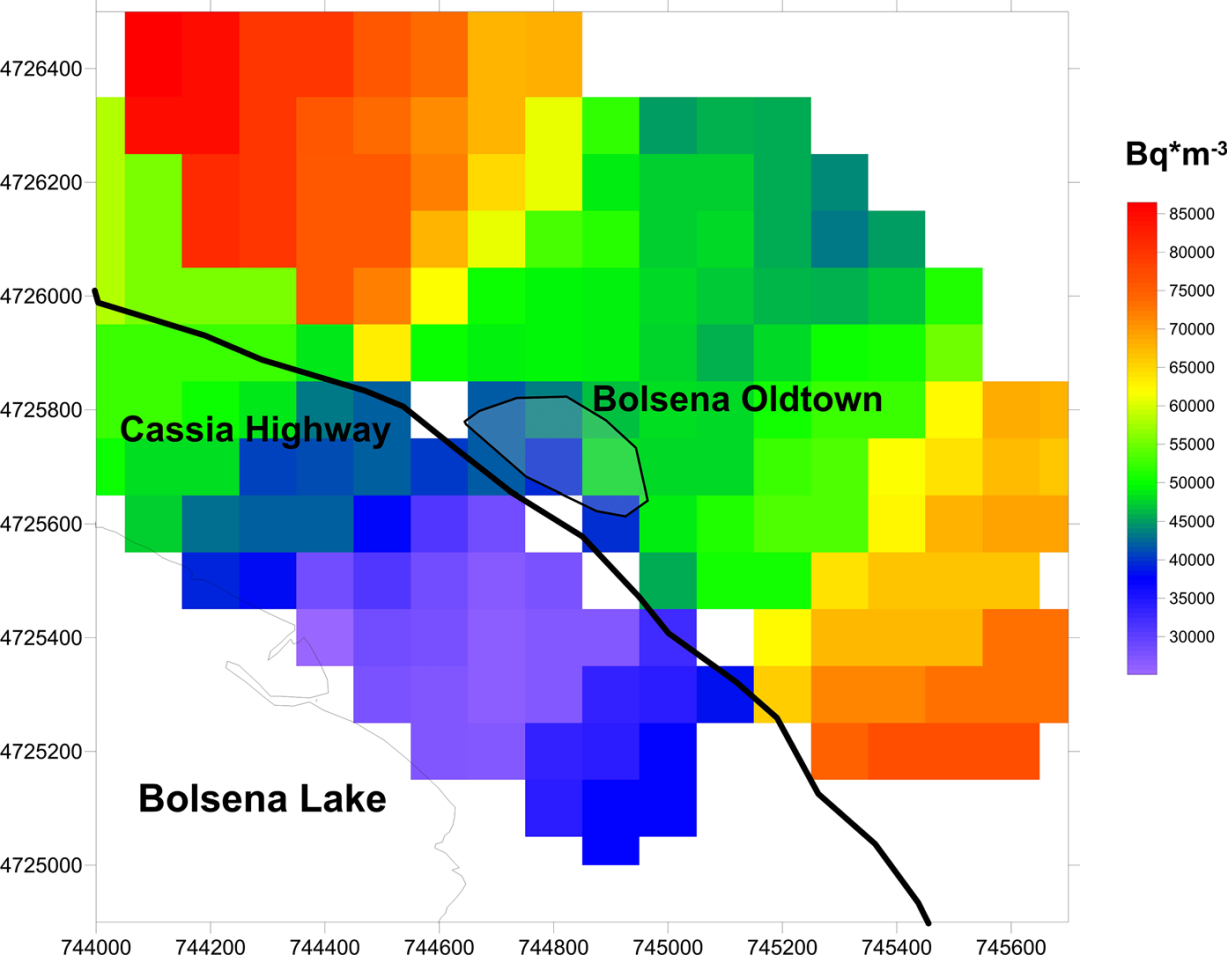
Map of soil gas radon concentrations in Bq/m^3^ based on geology and soil gas radon measurements

With this variogram model, soil gas radon maps were produced according to the methodology previously described in the “Radon mapping methodology”. Figure 7 shows the mean soil gas radon concentration over 0.1 × 0.1 km cells across the sampled area.

The graphical output of the geostatistical elaboration clearly shows that the spatial distribution of the soil gas radon data locates the lower concentration levels near the lake in correspondence of likely alluvial areas, while higher concentrations are located close to the old Bolsena village in correspondence with the volcanic outcrops, in agreement with previous comments.

### Radon risk assessment

**Fig. 8:**
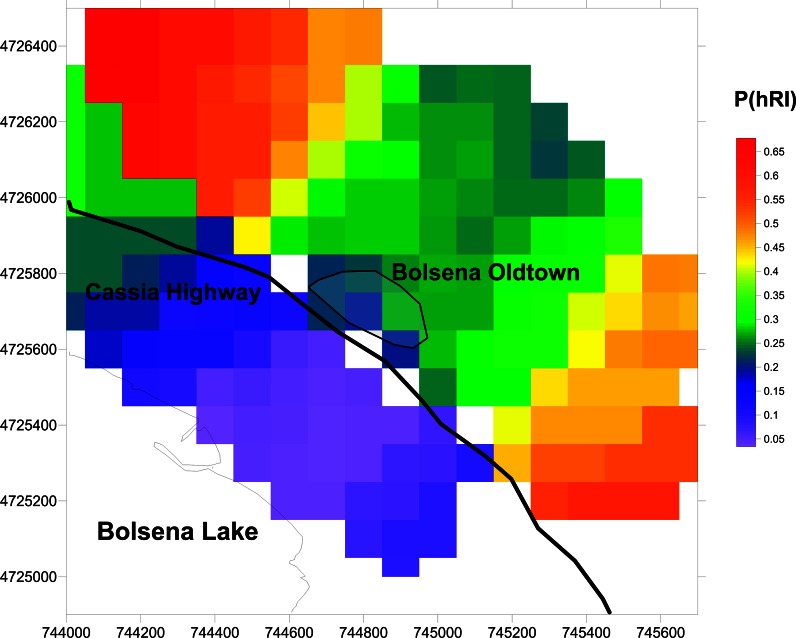
Map of probability to be in an area of high Radon Index—P(hRI) in  %

The radon-related information collected cumulatively in Bolsena and in the Volsini Volcanic district suggests the need to assess the level of radon risk in this region. The soil gas radon and permeability data collected were therefore employed to calculate the probability of being in an area with high Radon Index (defined in 3.5 and Table 1) considering separately the two geological groups, alluvial and volcanic products. The soils of the examined area were designed as medium permeable soils on the basis of the measurements of the weight percentage of the fine fraction carried out on the representative soil samples (thus *C*_*p*_ = 70 kBq/m^3^ in Eq. (1)). As a consequence of the constant variogram model, the standard deviation σ_g_ of the whole geological group can be applied to the local subgroups (10–15 data) used to establish the cell mean concentration. Figure 8 shows the probability of being in an area of high Radon Index (PhRI) over 0.1 × 0.1 km cells across the studied area. The area closer to the lake shows a PhRI below 30 %, while the volcanic area, in the upper part of the map, is characterized by probabilities ranging between 30 % and 70 %. White color indicates cells where in a distance (radius) of 500 m from cell center the code did not look up to find 10 data points to calculate the mean at the cell center.

The central part of the volcanic area presents lower values than the external part. Observing Fig. 1, it can be noted that while the central part of the sampling area is characterized by products coming from the Bolsena volcanic complex, the external part is described by volcanic products from the Orvieto volcanic complex. Considering data from Bolsena and, respectively, Orvieto volcanic complexes soil gas radon concentration mean values of 64 and 80 kBq/m^3^ can be found. Grouping together data measured in rocks coming from Bolsena and from Orvieto, the mean ^226^Ra activity concentrations are 187 and 231 Bq/kg, respectively (Capaccioni et al. 2012). Being the ^226^Ra the precursor of radon, the highest ^226^Ra activity concentration found in rocks from the Orvieto volcanic complex explains the higher value of soil gas radon concentrations in the lateral part (Orvieto complexes) and hence the higher percentage of high radon risk. We did not consider Bolsena and Orvieto as separate groups for mapping, because of the low number of data.

## Conclusions

A series of sixty-three soil gas radon measurements were carried out in Bolsena, the principal urban settlement in the Volsini Volcanic district, characterized by a higher than average natural radioactivity background in connection with Quaternary potassic volcanic lithologies.

Soil gas radon concentration ranges between 7 and 176 kBq/m^3^, indicating a large degree of variability in the NORM content and behavior of the parent soil material, related in particular to the occurrence of two different lithologies. The soil gas radon concentration measured in this study is consistent with the results of a calculation based on laboratory measurements of ^226^Ra and emanation factor, using the schematic diffusion of Nero (1988).

Soil gas radon mapping confirmed the existence of two different areas: lower soil gas radon area, close to the lake of Bolsena is consistent with an alluvial lithology, while close to the old Bolsena village, there is a dominance of the acidic high radioactive contribution of volcanic rocks from the Vulsini district with higher soil radon.

The result of the radon risk assessment, based on soil gas radon and permeability data, is a map where the alluvial area is characterized by a probability of being in an area with high Radon Index lower than 20 %, while the probability is higher than 30 % in the northernmost part of the map characterized by volcanic products, until reaching a probability above 50 % in the external areas described by products from the Orvieto volcanic complex.

Considering these results, as well as the high indoor radon building found in the same area (Capaccioni et al. 2012), an appropriate action should be undertaken to prevent radon pollution in new buildings. Our maps are a first sketch of the definition of the areas which should be protected.

## Footnotes

1 ICRP suggested (Publication 65, paragraph 76, 102) that Radon Prone Areas might be those parts of the country where at least 1 % of dwellings have radon levels more than ten times the national average as determined by appropriate statistical sampling (EC 1997).

2 NORM: acronym for Naturally Occurring Radioactive Materials.

3 More precisely, the Czech procedure applies to a given building site, and the radon concentration representative of the site is the third quartile of at least 15 measurements on the same site. Having done one single measurement per site, we take it as representative of the site.
